# Chromosome nondisjunction during bipolar mitoses of binucleated intermediates promote aneuploidy formation along with multipolar mitoses rather than chromosome loss in micronuclei induced by asbestos

**DOI:** 10.18632/oncotarget.14212

**Published:** 2016-12-26

**Authors:** Tianwei Zhang, Lei Lv, Yun Huang, Xiaohui Ren, Qinghua Shi

**Affiliations:** ^1^ Molecular and Cell Genetics Laboratory, The CAS Key Laboratory of Innate Immunity and Chronic Diseases, Hefei National Laboratory for Physical Sciences at Microscale, School of Life Sciences, CAS Center for Excellence in Molecular Cell Science, University of Science and Technology of China, Hefei 230027, Anhui, China; ^2^ Collaborative Innovation Center of Genetics and Development, Shanghai 200438, China

**Keywords:** asbestos, aneuploidy, binucleated cell, tetraploid, chromosome nondisjunction

## Abstract

Asbestos is a well-known occupational carcinogen that can cause aneuploidy during the early stages of neoplastic development. To explore the origins of asbestos-induced aneuploidy, we performed long-term live-cell imaging followed by fluorescence *in situ* hybridization of chromosomes 8 and 12 in human bronchial epithelial (HBEC) and mesothelial (MeT5A) cells. We demonstrate that asbestos induces aneuploidy via binucleated intermediates resulting from cytokinesis failure. On the one hand, asbestos increases chromosome nondisjunction during bipolar divisions of binucleated intermediates and produces near-tetraploidy. On the other hand, asbestos increases multipolar divisions of binucleated intermediates to produce aneuploidy. Surprisingly, chromosomes in asbestos-induced micronucleated cells are not truly lost by the cells, and do not contribute to aneuploid cell formation in either cell type. These results clarify the cellular source of asbestos-induced aneuploidy. In particular, they show the asbestos-induced disruption of bipolar chromosomal segregation in tetraploid cells, thereby demonstrating the causality between binucleated intermediates and aneuploidy evolution, rather than chromosome loss in micronuclei.

## INTRODUCTION

Asbestos is an established carcinogen that causes human malignancies, including malignant pleural mesothelioma (MPM), lung cancer, bronchial cancers, and various other cancers [1, 2]. The world-wide incidence of asbestos-associated cancers has been rising, mainly due to a long latency period of 10–30 years from the initial asbestos exposure to the development of illness [3, 4].

Aneuploidy, a hallmark of human cancers [5, 6], commonly results from chromosome missegregation including chromosome loss and nondisjunction [7–9], has been associated with asbestos-induced neoplastic development [10–12]. Chrysotile or crocidolite exposure associated chromosome instability (CIN) and consequent aneuploidy formation have been observed in various types of *in vitro* cultured mammalian cells [10, 13–19]. Furthermore, these numerical chromosome aberrations closely correlate with *in vitro* cell transformation [16–21]. However, how asbestos induces aneuploidy formation remains elusive.

During early stages of tumorigenesis, a transient tetraploid intermediate is formed, which, precedes the development of CIN and aneuploidy [22–26]. The unstable tetraploidy compromises the maintenance of genomic stability and facilitates the development of aneuploidy, cellular transformation, and tumor formation, frequently through chromosome missegregation during multipolar mitosis [22–24]. Interestingly, asbestos fibers can be trapped by the cleavage furrow and sterically block cytokinesis, resulting in the formation of binucleated cells [27–30]. In addition, multipolar mitosis and aneuploidy formation have been observed post asbestos treatment in fixed and living cells [13, 14, 30]. However, a direct linkage between binucleated cells, multipolar mitosis and aneuploidy induction, and whether possibly other pathways contributing to the formation of asbestos-induced aneuploidy remain unknown.

Chrysotile and crocidolite treatment directly interferes with spindle apparatus and chromosome behavior [20, 31], causing prevalent anaphase chromosomal abnormalities, such as lagging chromosomes and chromosomal bridges [15, 32, 33]. Correspondingly, a high frequency of micronucleus formation has been observed following chrysotile or crocidolite exposure [10, 34–36]. However, it remains to be elucidated whether micronucleated cells truly lose chromosomes and become aneuploid.

In the present study, we combined long-term live-cell imaging and fluorescence *in situ* hybridization (FISH) to investigate the mechanism of generation of aneuploid cells after asbestos treatment. Using this novel technique, we demonstrate the direct causality between binucleated cells induced by asbestos and aneuploidy formation. In addition to multipolar mitoses of binucleated cells as a main origin of aneuploidy, asbestos treatment significantly increases the chromosome nondisjunction rate during bipolar divisions of binucleated intermediates, which equally contributes to the aneuploid cell formation. However, chromosome loss in micronuclei is not the main contributor to asbestos-induced aneuploidy.

## RESULTS

### Asbestos treatment induces aneuploid cells

Immediate FISH analysis after long-term live-cell imaging was performed to examine the formation of aneuploid cells. In total, 2.89% (48/1661) of HBEC and 4.54% (37/815) of MeT5A daughter cells were observed as aneuploids. This was significantly higher (HBEC: *p* < 0.001, MeT5A: *p* < 0.001, 2 × 2 χ2 test) than in untreated groups (HBEC: 0.00%, MeT5A: 1.17%) (Table 1).

**Table 1:** Chrysotile treatment induces aneuploidy in cultured cell lines

Cell lines	Treatment	Number of cells analyzed	Number (%) of aneuploid cells for Chr 8 only	Number (%) of aneuploid cells for Chr 12 only	Number (%) of aneuploid cells for Chr 8 and 12	Total (%)
HBEC	Untreated	850	0 (0.00)	0 (0.00)	0 (0.00)	0 (0.00)
	Chrysotile A (2 μg/cm^2^)	1661	19 (1.14)*	25 (1.51)*	4 (0.24)	48 (2.89)*
MeT5A	Untreated	683	2 (0.29)	4 (0.59)	2 (0.29)	8 (1.17)
	Chrysotile A (2 μg/cm^2^)	815	9 (1.10)	21 (2.58)*	7 (0.86)	37 (4.54)*

Frequencies of aneuploid daughter cells were detected by FISH using centromeric probes specific to human chromosomes 8 and 12 following long-term live-cell imaging. Data are summarized from at least two independent experiments.

*p < 0.001, 2 × 2 χ^2^ test, compared with frequencies of aneuploidy in untreated cells.

### Asbestos induces binucleated cells through cytokinesis failure following elongated cytoplasmic bridge (CB) stage

**Figure 1:**
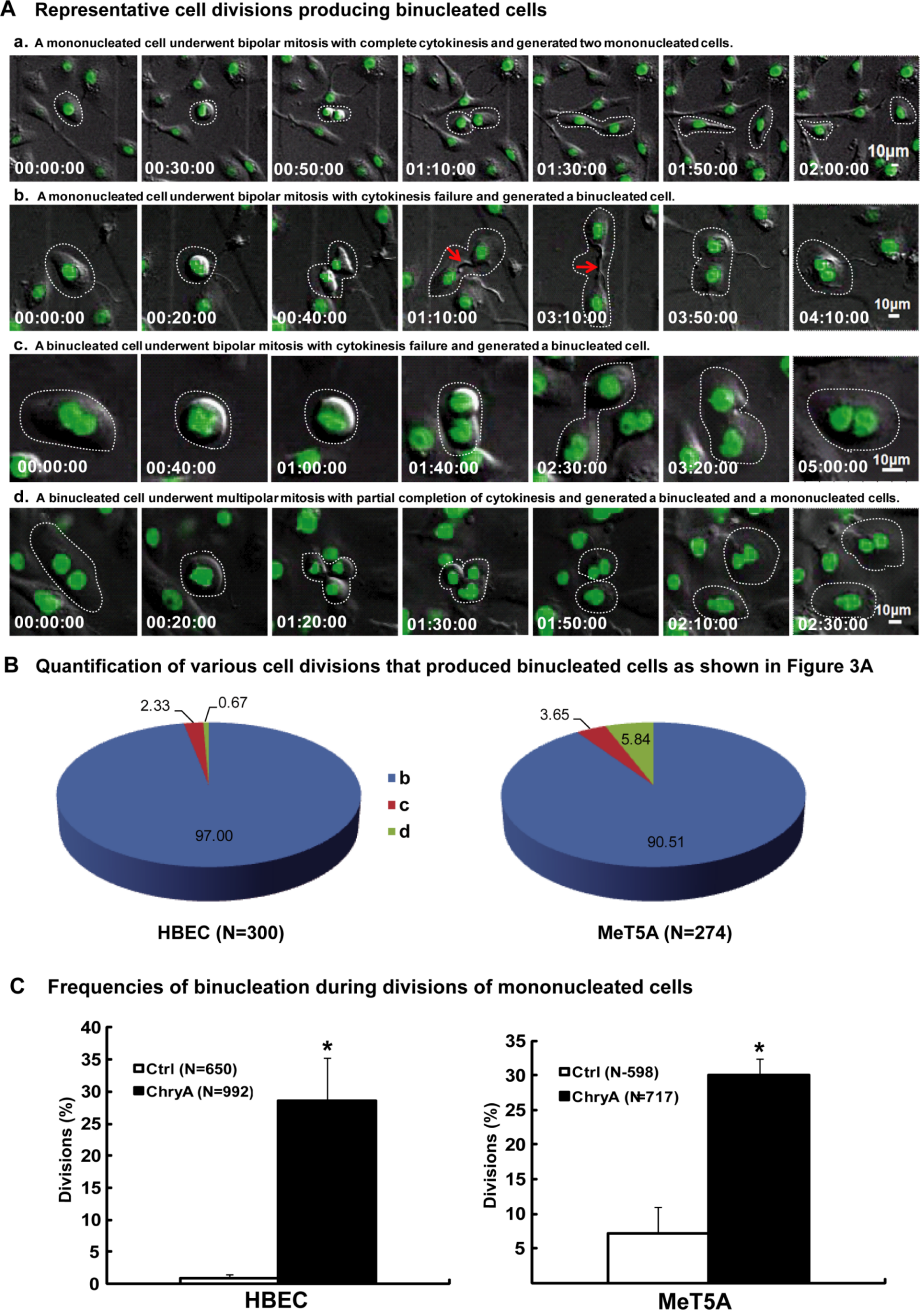
Asbestos induces binucleated cells through cytokinesis failure (**A**) Serial images showed representative HBEC cell normal division producing two mononucleated cells (Supplementary Movie S1) and divisions producing binucleated cells (Supplementary Movie S2–S4). Red arrows indicate asbestos across the cytoplasmic bridge region during divisions. Time is indicated in hours: minutes: seconds. (**B**) Quantification of various cell divisions producing binucleated daughter cells in HBEC and MeT5A cells after chrysotile treatment (N: the number of binucleated daughter cells analyzed). All the data were from at least two independent live-cell imaging experiments. (**C**) The frequency of binucleation in divisions was compared between untreated (Ctrl) and chrysotile-treated (ChryA) mononucleated HBEC and MeT5A cells (N: the number of divisions analyzed). *p < 0.001, 2 × 2 χ2 test.

We further examined and classified the origins of binucleated cells in chrysotile treated HBEC and MeT5A cells by live-cell imaging. Three origins were observed, including cytokinesis failure from mitoses of mononucleated cells, cytokinesis failure from mitoses of binucleated cells and incomplete multipolar mitoses (Figure 1A) (Supplementary Movie S1–S4). During the process of cytokinesis failure, the cytokinetic abscissions could not be completed and the cytoplasmic bridges regressed to produce binucleated cells (Figure 1A). Cytokinesis failure from mitoses of mononucleated cells was the main source of binucleated cells in both cell lines, producing 97.00 ± 4.06% (291/300) and 90.51 ± 4.47% (248/274) of binucleated daughter cells in chrysotile treated HBEC and MeT5A cells, respectively (Figure 1B).

As a further confirmation, we analyzed mitoses of mononucleated cells from live-cell imaging. Chrysotile-treated mononucleated HBEC and MeT5A cells had significantly increased frequency of binucleation (HBEC: 28.63 ± 6.69%; MeT5A: 29.99 ± 2.37%) compared to untreated cells (HBEC: 0.92 ± 0.61%, *p* < 0.001; MeT5A: 7.19 ± 3.80%, *p* < 0.001, 2 × 2 χ2 test) (Figure 1C). When these binucleated cells entered the next round of bipolar mitosis, even higher percentage of them continued to undergo cytokinesis failures and produced binucleated cells (HBEC: 32.38 ± 10.55%; MeT5A: 44.44 ± 7.93%), suggesting the dependence of binucleation on asbestos in the cells. *In vitro* experiments also confirmed that asbestos could induce high proportion of binucleated cells depending on the dosage and duration of treatment (Supplementary Figure S1).

Along with the high frequency of binucleation, elongation of cytoplasmic bridge (CB) stages was observed in chrysotile treated HBEC and MeT5A cells. The duration of CB stages in cells undergoing CB regression in chrysotile treated cells (HBEC: 164.79 ± 111.14 min; MeT5A: 353.58 ± 376.23 min) was longer than in cells undergoing CB abscission, no matter in untreated (HBEC: 111.33 ± 91.49 min, *p* < 0.001; MeT5A: 182.42 ± 93.11 min, *p* < 0.05; student's *t*-test) or chrysotile treated groups (HBEC: 111.25 ± 87.55 min, *p* < 0.001; MeT5A: 237.44 ± 77.50 min, *p* < 0.05; student's *t*-test) (Supplementary Figure S2).

### Asbestos induces aneuploid cells mainly through multipolar and bipolar divisions of binucleated cells

**Figure 2:**
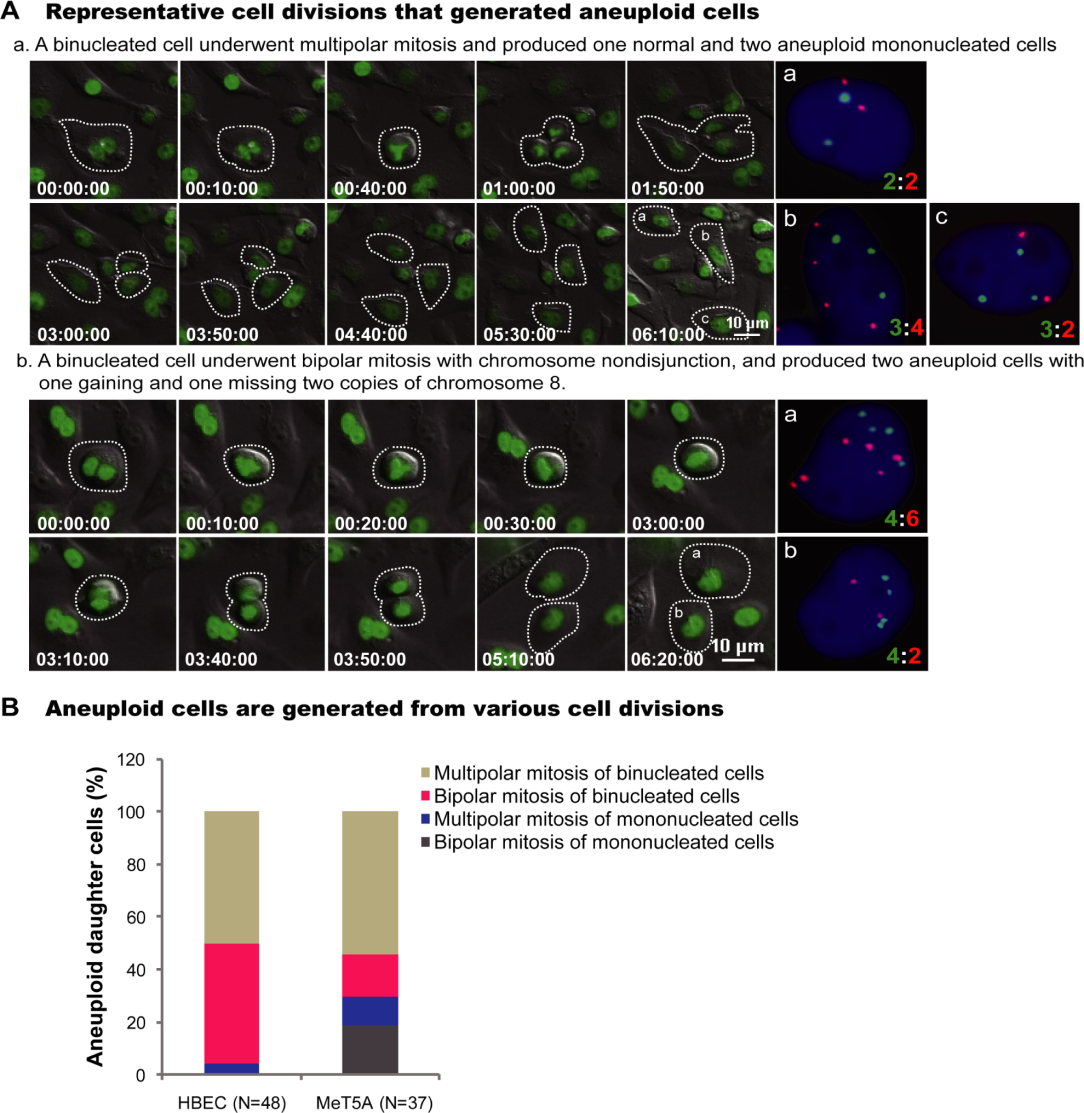
Representative cell divisions generating aneuploid cells (**A**) Serial images showed two representative HBEC cell divisions leading to aneuploid cells: (a) A binucleated cell underwent tripolar mitosis and produced three daughter cells with chromosome 12:8 compositions of 2:2, 3:4 and 3:2, respectively (Supplementary Movie S5); (b) A binucleated cell underwent bipolar mitosis with nondisjunction of chromosome 8, and produced two daughter cells with 4:6 and 4:2 for chromosome 12:8 (Supplementary Movie S6). Time is indicated in hours: minutes: seconds. The number of chromosomes in daughter cells presenting in the last frame of time-lapse imaging was directly assayed by FISH using chromosome 8 (Red) and 12 (Green) -specific probes immediately after live-cell imaging. (**B**) Quantification of cell divisions that produced aneuploid cells. N, the number of aneuploid daughter cells analyzed. Data were summarized from analysis of FISH signals following long-term live-cell imaging from at least two independent experiments.

The origins of all aneuploid daughter cells were then traced from the long-term live-cell imaging before FISH. The most common pathways for aneuploid cell generation were multipolar mitosis and bipolar divisions of binucleated cells (Figure 2A). For aneuploid HBEC cells, 46% (22/48) was from bipolar divisions of binucleated cells, 50% (24/48) was from multipolar divisions of binucleated cells, and only 4% (2/48) was from multipolar divisions of mononucleated cells (Figure 2B) (Supplementary Movie S5–S6). For aneuploid MeT5A cells, 16% (6/37) was from bipolar divisions of binucleated cells, 54% (20/37) was from multipolar divisions of binucleated cells, 19% (7/37) was from bipolar divisions of mononucleated cells, and 11% (4/37) was from multipolar divisions of mononucleated cells (Figure 2B). There was no significant difference in the frequencies of aneuploid daughter cell formation from mononucleated cells between untreated and chrysotile treated HBEC or MeT5A cells (HBEC: 0.00% vs 0.17%, *p* > 0.05; MeT5A: 0.88% vs 1.69%, *p* > 0.05; 2 × 2 χ2 test) (data not shown). These data indicate that the aneuploidy from divisions of mononucleated cells is independent of asbestos treatment, suggesting that the asbestos-induced aneuploidy is mainly attributed to the divisions of binucleated cells.

The aneuploidy frequency in daughter cells from mitoses of binucleated cells was 9.94% (46/462) in HBEC cells and 15.85% (26/164) in MeT5A cells. This is significantly higher than from mitoses of treated mononucleated cells (HBEC: 0.17% (2/1199), *p* < 0.001; MeT5A: 1.69% (11/651), *p* < 0.001; 2 × 2 χ2 test), also suggesting that binucleated cells are the origins of asbestos induced aneuploidy (Supplementary Figure S3).

### Asbestos induces chromosome nondisjunction during bipolar divisions of binucleated cells

To determine the effect of asbestos on divisions of binucleated cells, we used cytochalasinB (cytoB) induced binucleated cells as a control. FISH analysis was performed immediately after long-term live-cell imaging to examine the origin of aneuploidy formation from cytoB-induced binucleated HBEC and MeT5A cells. In total, 6.18% (22/356) of HBEC and 33.33% (87/261) of MeT5A aneuploid daughter cells were observed among all daughter cells divided from cyto-B induced binucleated cells. Tracing back to examine the aneuploidy formation pathway, we observed that cytoB-induced binucleated cells could enter both bipolar mitosis (HBEC: 89.96%; MeT5A: 42.78%) and multipolar mitosis (HBEC: 10.14%; MeT5A: 57.22%), which was similar to the asbestos-induced binucleated cells (bipolar mitosis: 82.17 ± 2.06% in HBEC, 54.14 ± 13.51% in MeT5A; multipolar mitosis: 17.83 ± 2.06% in HBEC, 45.86 ± 13.51% in MeT5A). All aneuploid daughter cells from cytoB-induced binucleated cells were from multipolar mitosis (HBEC: 22/22; MeT5A: 87/87), while a large proportion of aneuploid cells from asbestos-induced binucleated cells were from bipolar mitosis (HBEC: 22/46; MeT5A: 6/26), indicating that asbestos treatment could directly interrupt normal chromosome segregation during bipolar divisions.

**Figure 3:**
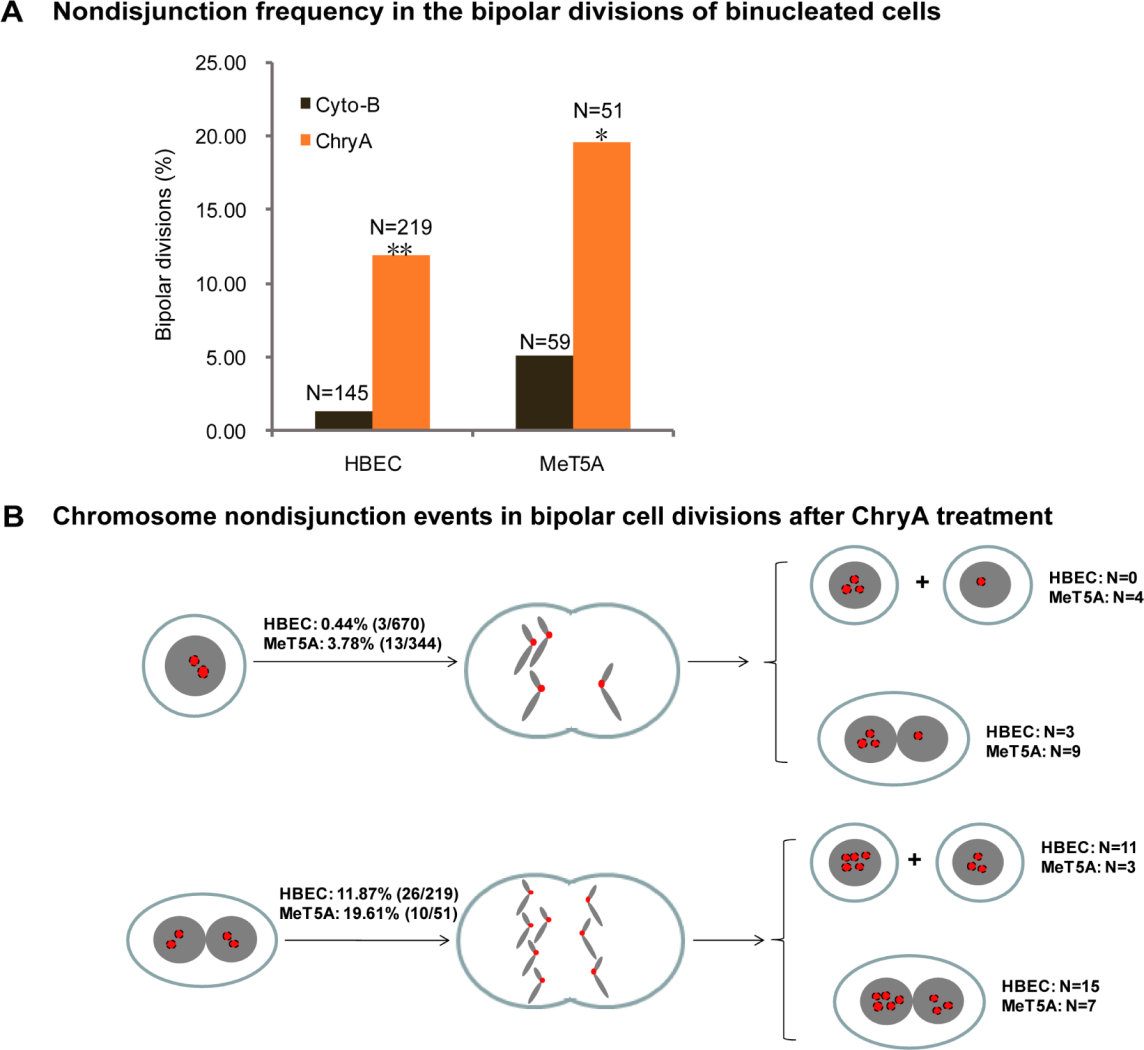
Asbestos increases frequency of chromosome nondisjunction in the bipolar divisions of binucleated cells (**A**) The frequency of chromosome nondisjunction was compared in the bipolar divisions of cytochalasin-B induced (Cyto-B) and chrysotile-induced (ChryA) binucleated cells (N: the number of bipolar divisions analyzed). *p < 0.05, **p < 0.001, 2 × 2 χ2 test. The number of chromosomes in daughter cells presenting in the last frame of time-lapse imaging was directly assayed by FISH using chromosome 8 (Red) and 12 (Green) -specific probes immediately after live-cell imaging from at least two independent experiments. (**B**) A schematic diagram summarizing the chromosome nondisjunction events in the bipolar divisions of binucleated HBEC and MeT5A cells after chrysotile treatment (N: the number of bipolar divisions analyzed).

Thus, we examined the fidelity of chromosome segregation during bipolar divisions of asbestos-induced binucleated cells by analyzing the copies of chromosomes 8 and 12 in daughter cells. 11.87% (26/219) of bipolar divisions in binucleated HBEC cells and 19.16% (10/51) of bipolar divisions in binucleated MeT5A cells experienced chromosome nondisjunction (Figure 3A). This was significantly higher compared to that in cytoB-induced binucleated cells in both cell lines (HBEC: 11.87% vs. 1.38% (2/145), *p* < 0.001; MeT5A: 19.61% vs. 5.08% (3/59), *p* < 0.05, 2 × 2 χ2 test) (Figure 3A). In addition, it was higher than in asbestos-treated mononucleated cells (HBEC: 11.87% vs. 0.44% (3/670), *p* < 0.001; MeT5A: 19.16% vs. 3.78% (13/344), *p* < 0.001, 2 × 2 χ2 test) (Figure 3B), and untreated mononucleated cells (HBEC: 11.87% vs. 0.00% (0/421), *p* < 0.001; MeT5A: 19.16% vs. 0.90% (3/333), *p* < 0.001, 2 × 2 χ2 test). Consequently, 42% (11/26) of HBEC daughter cells and 30% (3/10) of MeT5A daughter cells generating from these chromosome nondisjunction events were near-tetraploid mononucleated cells (Figure 3B).

### Chromosomes in asbestos-induced micronucleated cells are not truly lost by the cells

**Figure 4:**
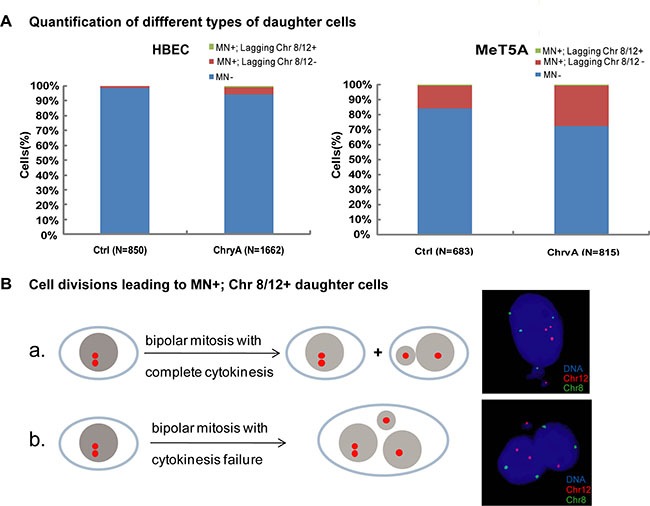
Chromosome loss through micronuclei after chrysotile treatment does not generate aneuploid cells (**A**) Quantification of different types of daughter cells in untreated and chrysotile-treated (ChryA) HBEC and MeT5A cells. (MN+) indicates micronuclei-bearing cells, (MN+; chr 8/12-) indicates micronuclei-bearing cells without chromosome 8 or 12 FISH signals in the micronuclei, while (MN+; chr 8/12+) indicates micronuclei-bearing cells with chromosome 8 or 12 FISH signals in the micronuclei (N: the number of daughter cells analyzed). (**B**) A schematic diagram showed two types of divisions in HBEC cells resulting in micronucleated daughter cells with chromosome 8 or 12 FISH signals in the micronuclei (MN+; chr 8/12+): (a) the chromosome was distributed into the right daughter cell along with the micronucleus; (b) daughter cells fused and formed a binucleated or multinucleated cell, both of which types generating euploid cells as shown by the representative FISH images (right). All the data were summarized from FISH following long-term live-cell imaging from at least two independent experiments.

Previous studies have suggested that chromosome loss through micronucleation might contribute to asbestos-induced aneuploidy. Thus, we examined the loss of specific chromosomes (chromosomes 8 and 12) during micronucleation from the mitoses of mononucleated cells from live-cell imaging analysis. Most daughter cells after chrysotile-treatment showed no micronucleation (MN-), or no chromosome 8 or 12 loss in the micronucleus (MN+; Chr 8/12-); while only 0.60% (10/1661) of HBEC and 0.61% (5/815) of MeT5A cells contained chromosome 8 or 12 signal in the micronucleus (MN+, Chr 8/12+) (Figure 4A). This was much lower than the frequencies of aneuploidy formation with chromosome 8 or 12 abnormalities (HBEC: 2.89%; MeT5A: 4.54%). In addition, by analyzing chromosome distribution in daughter cells with chromosome 8 or chromosome 12 in micronucleus, we found that all micronuclei-bearing cells with lost chromosome 8 or 12 (MN+; Chr 8/12+) could be categorized into two types, which were not aneuploid. Either the chromosome was distributed into the right daughter cell along with the micronucleus; or the daughter cells fused and formed a binucleated or multinucleated cell (Figure 4B). In the present study, all aneuploidy was found in the main nucleus of cells. Our results suggest that chromosomes in asbestos-induced micronucleated cells are not truly lost by the cells, and are not directly contributing to the aneuploidy formation.

**Figure 5:**
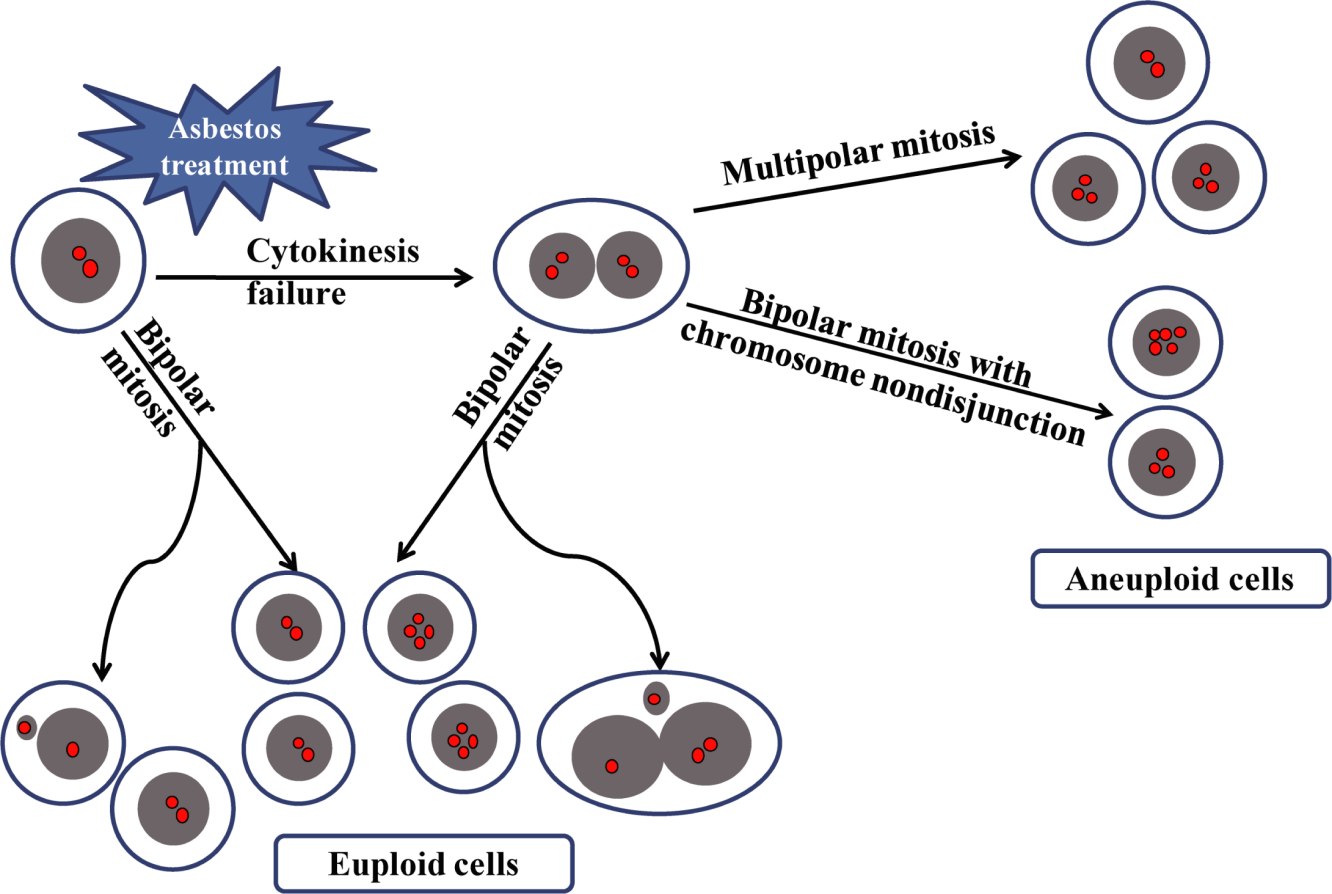
A schematic diagram summarizing the origin of aneuploid cells induced by asbestos Cytokinesis failure of mononucleated cellsbecause of asbestos treatment leads to binucleated cells, which produced aneuploid cells through either multipolar mitosis or bipolar mitosis with chromosome nondisjunction. While micronucleated cells resulted from bipolar mitosis of mononucleated or binucleated cells do not contribute to the generation of aneuploid cells.

Together, we combined long-term live cell imaging and FISH technique to reveal the origins of aneuploidy formation induced by asbestos. As shown in the schematic diagram in Figure 5, we demonstrate that asbestos-induced binucleated intermediate cells produce aneuploid progenies. Not surprisingly, multipolar divisions of binucleated cells contribute to aneuploidy formation. Furthermore, increase of chromosome nondisjunction during the bipolar divisions of binucleated cells is induced by asbestos treatment, which plays an important role in aneuploidy formation. In contrast, chromosomes in asbestos-induced micronucleated cells are not truly lost by the cells, and do not contribute to aneuploidy formation.

## DISCUSSION

Asbestos is an occupational carcinogen for human malignancies, and is especially related to malignant mesotheliomas and lung cancers [1, 2]. In this study, we used two immortalized cell lines, human bronchial epithelial cell line HBEC and human mesothelial cell line MeT5A, as models for respiratory tumors and malignant mesotheliomas that frequently originate from these two cell types, respectively. Chrysotile is characterized by curves and silken fibers; it comprises over 95% of asbestos used around the world. Although considered less harmful to human health possibly because of its faster clearance from tissues and less accumulation *in vivo* [37–39], the carcinogenicity of chrysotile has been fully confirmed together with other types of asbestos fibers [40, 41].

Aneuploidy, a hallmark of cancer, is commonly found in asbestos associated cancers [10–12]. *In vitro* studies demonstrated correlation between aneuploidy formation post asbestos exposure and chromosome aberrations [16–21], and multipolar mitoses increase [13, 14, 30]. However, these correlations were commonly deducted from observations by individual detection methods, including metaphase chromosome analysis, DNA quantification by Feulgen's reaction and time-lapse microscopy. The disadvantages of these conventional methods are that they cannot precisely classify the origins of aneuploidy, or assess the fidelity of chromosome segregation. Therefore, we performed fluorescence *in situ* hybridization (FISH) following long-term live cell imaging to examine the direct causalities between asbestos exposure, chromosome segregation, and aneuploidy formation. This novel approach has several advantages compared to conventional methods. First, chromosome loss (FISH signals appeared in micronuclei) and nondisjunction can be precisely identified simultaneously with chromosome specific centromeric DNA probes for FISH. Second, by analyzing live cell imaging before FISH, overestimation of the frequency of aneuploidy in fixed cells because of signal artifacts (for example, close, overlapping, missing or split signals) can be avoided. Third, we can trace how euploidy evolved into aneuploidy by live cell imaging, which provides a more accurate evidence for aneuploidy induction by asbestos. For FISH, probes targeting chromosomes 8 and 12 were chosen based on the following reasons: They are from the Group C of human chromosomes and of medium size. The results generated are more representative for common chromosome segregation occurrence in cells. Our previous results have indicated that the chromosome missegregation rates for autosomes are similar in human cell lines [22, 42, 43]. In addition, chromosome specificity and brightness of FISH signals are much better than other FISH probes we tested.

Our study showed a major role of binucleated intermediates cells in the asbestos- induced aneuploidy, which generated 96% of aneuploid daughter cells in HBEC cells and 70% in MeT5A cells (Figure 2 and Supplementary Figure S1). This result provides a strong evidence for the causative role of transient tetraploid intermediates during development of aneuploidy and carcinogenesis [22–26]. Furthermore, consistent with previous studies demonstrating an increase of multipolar mitosis in chrysotile-treated cells [13, 14, 30], by tracking from live cell imaging we observed that 50% of aneuploid HBEC cells and 54% MeT5A cells were from multipolar divisions of binucleated intermediates (Figure 2). This confirmed multipolar mitoses of tetraploid intermediates as one of the most important origins of aneuploidy [22–24]. Interestingly, most of the aneuploid cells from multipolar mitoses of binucleated intermediates were mononucleated cells (15/24 in HBEC cells, and 12/20 in MeT5A cells). This supports the view that mononucleated cells inheriting suitable genome composition from de-polyploidization of polyploid cells might have a better potential for long-term survival than binucleated or multinucleated cells [44–46].

Importantly, we found that asbestos could directly damage the fidelity of chromosome segregations during bipolar divisions in binucleated cells. Consequently, 46% of aneuploid HBEC cells and 17% of MeT5A cells, which were all near-tetraploid mononucleated cells, were from bipolar divisions of asbestos-induced binucleated cells (Figure 2). Thus, bipolar mitosis of asbestos-induced binucleated cells might have an equally fateful contribution to the accumulation of aneuploidy and the phenotypic changes of populations due to the more pervasive occurrence and more viable progenies. Furthermore, significantly increased chromosome nondisjunction frequencies were observed during bipolar divisions of asbestos-induced binucleated cells, compared to both asbestos-treated mononucleated cells and cytoB-induced binucleated cells (Figure 3). The higher frequency of chromosome nondisjunction in binucleated cells than in mononucleated cells might be caused by the fact that most mononucleated cells did not contain asbestos fibers in the cytoplasm during divisions, while binucleated cells had a higher probability to have asbestos fibers trapped in the cytoplasm at the cytoplasmic bridge region during cytokinesis stage. Together, our results demonstrate a direct interference of asbestos with the fidelity of chromosome segregation during bipolar divisions of binucleated cells.

The correlation between asbestos-induced chromosome loss [15, 32, 33], micronuclei [10, 34–36], and aneuploidy has been suggested in *in vitro* cultured cell lines. Several studies have shown that asbestos increased micronuclei with chromosomal fragments but not whole chromosomes [10, 35]. However, because of technical limitations of studies on fixed cells, it was not examined whether these micronucleated cells were aneuploid, and the dynamic of chromosome loss was unclear. In the present study, we confirmed that the frequency of specific chromosome loss in micronuclei was much lower than the corresponding frequency of aneuploidy induction (Figure 4A). Furthermore, we found that all “lost” chromosomes were distributed into the right daughter cells (Figure 4B), so that the cell karyotype would recover to normal level when the micronucleated cells entered the next round of mitosis, as was also confirmed in cultured Hela cells [43]. Thus, our data demonstrated that chromosomes were not truly lost by the cells and that the micronucleated cells were indeed not aneuploid.

Taken together, we confirmed previous deductions that asbestos exposure induces binucleated intermediates, which promote aneuploidy formation through multipolar divisions. Furthermore, we demonstrate that asbestos can directly induce chromosome nondisjunction during bipolar divisions of the binucleated intermediate cells, as an equally important pathway for aneuploidy evolvement. In contrast to conventional assumption, chromosome loss in micronuclei caused by asbestos treatment does not substantially contribute to aneuploidy formation. Collectively, our results clarify the origins of asbestos-induced aneuploidy, identify the significant role of binucleated cells during the development of asbestos- induced aneuploidy, and demonstrate the direct effect of asbestos on the fidelity of chromosome segregations in tetraploid cells.

## MATERIALS AND METHODS

### Preparation of asbestos fibers

Chrysotile A Rhodesian Asbestos (SPI supplies #02701-AB, West Chester, USA) of UICC standard was purchased from SPI supplies. Stock solutions of chrysotile were prepared at 500 μg/ml of dry weight in phosphate buffered saline (PBS), dispersed by sonication and autoclaved (121°C, 30 min).

### Cell culture

Euploid human bronchial epithelial cells (HBEC) immortalized by expressing hTERT and Cdk4 [47], were provided by Dr. Minna (the University of Texas Southwestern Medical Center, Dallas, Texas, United States), and cultured in keratinocyte-SFM medium (Gibco #10724-011, Carlsbad, California, United States) with supplements for keratinocyte-SFM (Gibco #37000-015, Carlsbad, California, United States). MeT5A, an SV40-immortalized human mesothelial cell line was purchased from ATCC (#CRL-9444, Manassas, VA, USA) and cultured in complete growth medium 199 (Gibco #31100-035, Carlsbad, CA) as ATCC recommended. The medium contained 0.75 mM glutamine, 1.25 g/L sodium bicarbonate, 3.3 nM epidermal growth factor (EGF, Invitrogen #13247-051, CA, USA), 400 nM hydrocortisone, 870 nM insulin, 20 mM HEPES, 10% (vol/vol) fetal bovine serum (HyClone #SV30087.02, Thermo Fisher Scientific, MA), 100 U/ml penicillin (Gibco #15140-122), and 100 μg/ml of streptomycin (Gibco #15140-122). All incubations were performed at 37°C in a humidified atmosphere containing 5% CO_2_.

### Establishment of H2B-GFP expressing cells

Cells stably expressing H2B-GFP were obtained by retrovirus infection and micromanipulation. Firstly, a retroviral vector (pL-H2BGFP) and a packaging vector (PIK) were cotransfected into a packaging cell line (293FT) using Lipofectamine 2000 transfection reagent (Invitrogen #11668-027). Forty-eight hours after transfection, ecotropic retroviral supernatants were collected by centrifugation. Then, HBEC and MeT5A cells were infected with medium containing retroviral supernatant in the presence of 4 μg/ml of Polybrene Transfection Reagent (Millipore, #TR-1003, Billerica, Massachusetts, United States) for 12 hours, and then recovered in fresh medium for 24 hours. The cells expressing H2B-GFP were picked by micromanipulation and cultured without drug selection.

### *In vitro* binucleation assay

To analyze the effect of asbestos on binucleation, HBEC and MeT5A cells were seeded on coverslips in 60 mm culture dishes for 24 hours and treated with chrysotile or crocidolite at doses of 0, 2, 5, and 10 μg/cm^2^. The coverslips were taken out at 24, 48, 72, 96 hours (HBEC cells), or 30, 60, 90, 120 hours (MeT5A cells), rinsed in PBS and fixed in methanol: acetic acid (3:1 v/v) at –20°C for 20 min. For analysis, the coverslips were rinsed in PBS, stained by Diff-Quick cell stain, and counted by using a Leica light microscope (Wetzlar, Germany).

### Live cell imaging

Cells were seeded in a 35 mm glass bottom dish (MatTek Corporation, Ashland, MA) and incubated at 37°C in a humidified 5% CO_2_ containing atmosphere. Twenty-four hours after seeding, chrysotile was added into the medium at a dose of 2 μg/cm^2^ preceding immediate live cell imaging without washout during the live cell imaging. Images were acquired automatically using a Nikon TE2000E inverted microscope equipped with the Nikon Perfect Focus system (Nikon, Tokyo, Japan), a linearly-encoded stage (Proscan, Prior Scientific Corporation, Cambridge, London, UK) and a cooled CCD camera (Orca ER, Hamamatsu, Japan). The microscope was controlled using NIS-Elements Advanced Research (Nikon, Tokyo, Japan) software and housed in a custom-designed 37°C chamber with a secondary internal chamber that delivered humidified 5% CO2. Fluorescence illumination was generated by a mercury-arc lamp with two neutral density filters (for a total 64-fold reduction in intensity). Green fluorescent (GFP) and differential interference contrast (DIC) images were captured at multiple locations every 10 min (HBEC cells) or 20 min (MeT5A cells) for a period of 48–72 hours (48 hr for HBEC cells; 72 hr for MeT5A cells) or 18–46 hours (cytochalasin B group) with a ×20 Plan Apo objective. Images of incubated cells without treatment were also acquired as control. In cytochalasin B treatment experiments, cells were treated with 1 μg/ml cytochalasin B for 24 hours, washed, and subjected to live cell imaging. Immediately after live cell imaging, cells were fixed in methanol: acetic acid (3:1 v/v) at –20°C for fluorescence *in situ* hybridization (FISH). When performing FISH, the coverslips of culture dishes were removed from the dishes by soaking in Dow Corning fluid OS30 (MatTek Corporation) and washing in ddH2O.

For analysis of live cell imaging movies, the time-lapse records of live cell imaging experiments were exported as image series, and analyzed manually using NIS-Elements Advanced Research (Nikon, Tokyo, Japan) software. The criteria of analysis were as described previously [48]. Briefly, cytoplasmic bridge abscission, the final step of cytokinesis, was identified by the breakage of intercellular cytoplasmic bridge and complete separation of individual daughter cells. Cleavage furrow regression, the final step of cytokinesis failure, was identified by the disappearance of intercellular cytoplasmic bridge and beginning of cytoplasmic fusion of daughter cells. Cytoplasmic bridge (CB) stage was defined as the timing from cleavage furrow ingression to completion of abscission or furrow regression. Micronuclei were identified as the extra-nuclear green fluorescent-positive bodies with size less than 1/3rd of the main nuclei [49]. Cells were identified to undergo cell cycle arrest when they were observed not to enter into mitosis within one and a half cell cycles.

### Fluorescence *in situ* hybridization (FISH)

Plasmids encoding chromosome-specific centromeric probes were obtained from ATCC (Chromosome 8: pJM128, #61398; Chromosome 12: pA12H8, #59904, Manassas, VA, USA). Plasmid DNA was labeled with SpectumRed dUTP (Vysis #30-803400, IL, USA) or SpectrumGreen dUTP (Vysis #30-803200) using a nick translation system (Invitrogen #18160-010, Carlsbad, CA, USA).

Cells on coverslips were washed by ddH2O, affixed to microscope slides, and incubated sequentially in 2 × SSC 30 min, 1% PFA in PBS 10 min and 0.1% NP-40 in 2 × SSC 10 min each at room temperature. Slides were then incubated in increasing concentrations of ethanol (80%–90%–100%) for 2 min, respectively. After an open-air drying, 15 μl of hybridization solution containing respectively 2.25 μl human centromeric probes for chromosome 8 and 12 and 10.5 μl hybridization buffer were added to each coverslip. Coverslips were sealed by a new cover-glass and heated at 82°C for 8 min on a hot plate, then shifted to a humidified chamber and incubated at 37°C for 24 hours. The top cover-glass was removed and slides were washed in 2 × SSC at 45°C for 30 min. Nuclei were stained in 100 ng/ml Hoechst 33342 for 10 min and rinsed once in PBS. The cells were covered with a new cover-glass in Vectashield mounting medium (Vector Laboratories, #H-1000, Burlingame, CA, USA).

FISH Slides were examined using an Olympus BX-61 fluorescence microscope fitted with band pass filters (Olympus, Tokyo, Japan) detecting Hoechst, SpectrumRed and SpectrumGreen. Images were acquired with a cooled CCD camera operated by Image Pro Plus software (Media Cybernetics, MD, USA). For analysis of FISH, nuclei were scored as having two or more copies of a specific chromosome if the signals of the same color were of similar size and intensity and separated by a distance of more than half the diameter of the spot. For daughter cells from one mitosis following live cell imaging, to eliminate artifacts (for example, close, overlapping, missing or split signals), only cells coming from an euploid parental cell and having an even total number of hybridization signals from all daughter cells for every chromosome were scored.

### Statistical analysis

The Student's *t*-test was used to compare continuous variables and the Chi-square (χ2) test was used to compare categorical variables. The *p*-values < 0.05 were considered as statistically significant.

## SUPPLEMENTARY MATERIALS FIGURES AND MOVIES














