# Supplementary material for: Chromosome nondisjunction during bipolar mitoses of binucleated intermediates promote aneuploidy formation along with multipolar mitoses rather than chromosome loss in micronuclei induced by asbestos
Source: Oncotarget. 2016 Dec 26;8(7):11030–41. doi: 10.18632/oncotarget.14212 (PMC5355243; doi:10.18632/oncotarget.14212)
Supplement: Supplementary file 1 [file oncotarget-08-11030-s001.pdf]

Chromosome nondisjunction during bipolar mitoses of binucleated intermediates promote aneuploidy formation along with multipolar mitoses rather than chromosome loss in micronuclei induced by asbestos

Supplementary Materials

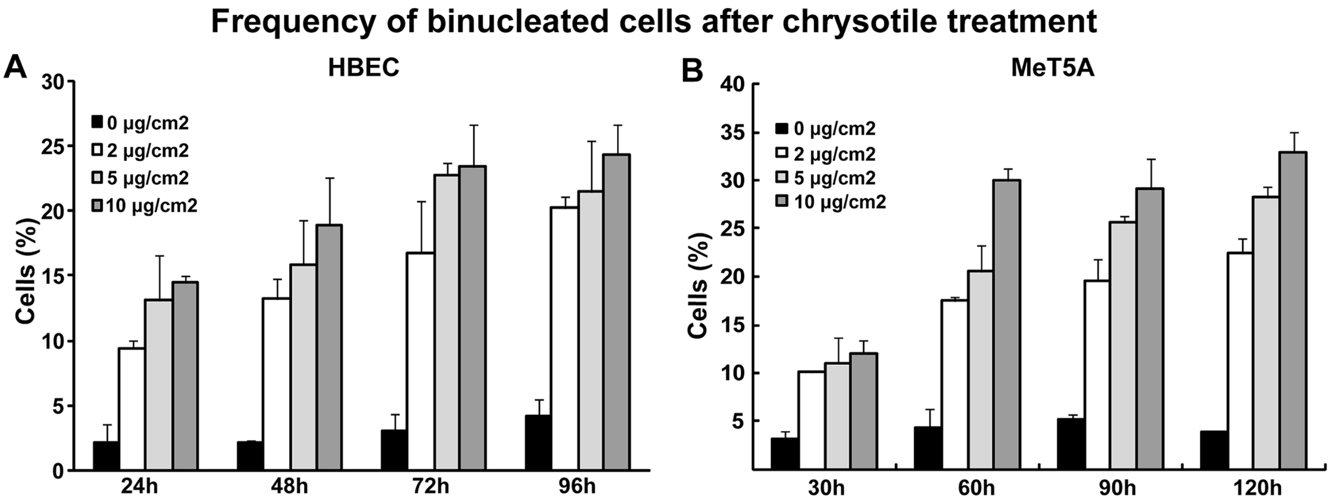

Supplementery Figure S1: Chrysotile treatment induces a time- and concentration-dependent increase of binucleated cells. The percentage of binucleated cells in the population were analyzed at 24, 48, 72, 96 hours (HBEC cells) or 30, 60, 90, 120 hours (MeT5A cells) after 0, 2, 5, 10µg/cm^2 chrysotile treatment. Two independent experiments were performed and more than 500 cells were analyzed in each experiment.

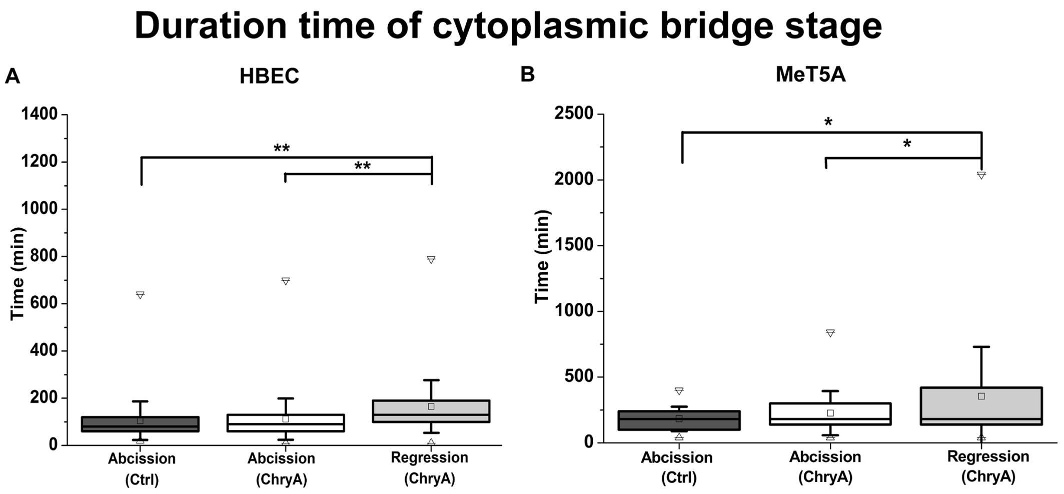

Supplementery Figure S2: Asbestos treatment prolongs the duration of cytoplasmic bridge stage during mitosis. The duration time of cytoplasmic bridge stages were compared between chrysotile-treated cells undergoing cleavage furrow regression (Regression (ChryA)) or cytoplasmic bridge abscission (Abcission (ChryA)) and untreated cells undergoing cytoplasmic bridge abscission (Abcission (Ctrl)). Cytoplasmic bridge abscission, the final step of cytokinesis, was identified by the breakage of intercellular cytoplasmic bridge and complete separation of individual daughter cells. Cleavage furrow regression, the final step of cytokinesis failure, was identified by the disappearance of intercellular cytoplasmic bridge and beginning of cytoplasmic fusion of daughter cells. Cytoplasmic bridge (CB) stage was defined as the timing from cleavage furrow ingression to completion of abscission or furrow regression. Data were analyzed from live-cell imaging. *$p < 0.05$, **$p < 0.001$, *t*-test.

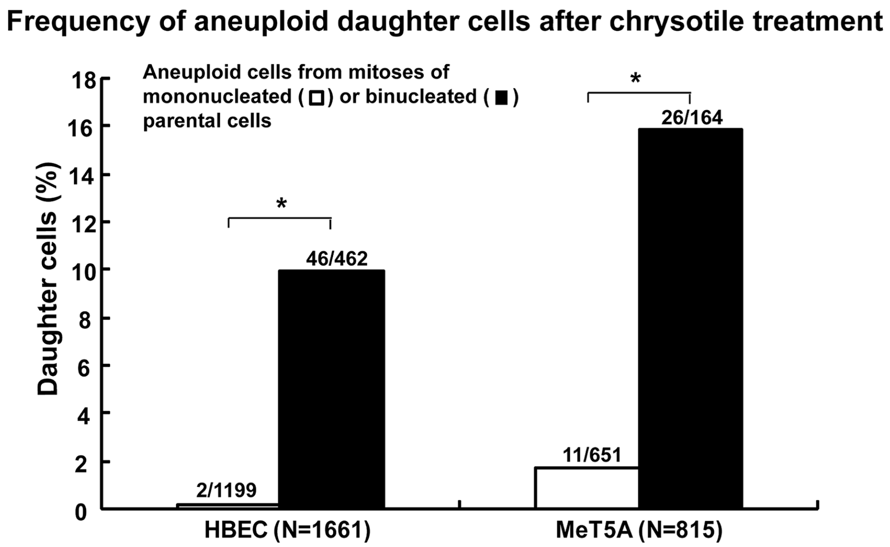

Supplementery Figure S3: Frequency of aneuploid daughter cells from different types of parental cells after chrysotile treatment. The frequency of aneuploid daughter cells generated from chrysotile-induced binucleated cells was compared with that from mononucleated cells after chrysotile treatment. *$p < 0.001$, 2×2 χ^2 test. Aneuploid daughter cells were detected by FISH using centromeric probes specific to human chromosome 8 and 12 immediately following long-term live-cell imaging. Data are summarized from from at least two independent long-term live-cell imaging experiments (N: the number of daughter cells analyzed).

Supplementary Movie S1: A normal mitosis completing cytokinesis and generating two mononucleated HBEC cells. See Supplementary_Movie_S1

Supplementary Movie S2: Cytokinesis failure in bipolar mitosis of one mononucleated HBEC cell generating one binucleated cell. See Supplementary_Movie_S2

Supplementary Movie S3: Cytokinesis failure in bipolar mitosis of one binucleated HBEC cell generating one binucleated cell. See Supplementary_Movie_S3

Supplementary Movie S4: Incomplete multipolar mitosis generating binucleated HBEC cell. See Supplementary_Movie_S4

Supplementary Movie S5: A binucleated HBEC cell producing aneuploidy from multipolar mitosis. See Supplementary_Movie_S5

Supplementary Movie S6: A binucleated HBEC cell producing aneuploidy from bipolar mitosis. See Supplementary_Movie_S6
